# Development of a nomogram for predicting grade 2 or higher acute hematologic toxicity of cervical cancer after the pelvic bone marrow sparing radiotherapy

**DOI:** 10.3389/fpubh.2022.993443

**Published:** 2022-09-09

**Authors:** Xiangdi Meng, Nan Wang, Meng Yu, Dechen Kong, Zhengtao Zhang, Xiaolong Chang, Yinghua Guo, Yang Li

**Affiliations:** ^1^Department of Radiation Oncology, Weifang People's Hospital, Weifang, China; ^2^Clinical School, Weifang Medical University, Weifang, China

**Keywords:** cervical cancer, intensity-modulated radiation therapy, nomogram, hematologic toxicity, risk stratification

## Abstract

**Background:**

Acute hematologic toxicity (HT) is a common complication during radiotherapy of cervical cancer which may lead to treatment delay or interruption. Despite the use of intensity-modulated radiation therapy (IMRT) with the pelvic bone marrow (PBM) sparing, some patients still suffer from acute HT. We aimed to identify predictors associated with HT and develop a nomogram for predicting grade 2 or higher (G2+) acute HT in cervical cancer following the PBM sparing strategy.

**Methods:**

This study retrospectively analyzed 125 patients with cervical cancer who underwent IMRT with the PBM sparing strategy at our institution. Univariate and multivariate logistic regression, best subset regression, and least absolute shrinkage and selection operator (LASSO) regression, respectively, were used for predictor screening, and Akaike information criterion (AIC) was used to determine the best model for developing the nomogram. Finally, we quantified the risk of G2+ acute HT based on this model to establish a risk stratification.

**Results:**

The independent predictors used to develop the nomogram were histological grade, pre-radiotherapy chemotherapy, pre-radiotherapy HT, and radiotherapy [IMRT alone vs. concurrent chemoradiotherapy (CCRT)] which were determined by the univariate and multivariate logistic regression with the minimum AIC of 125.49. Meanwhile, the heat map showed that there is no multicollinearity among the predictors. The nomogram was well-calibrated to reality, with a Brier score of 0.15. The AUC value was 0.82, and the median Brier score and AUC in 1000 five-fold cross-validation were 0.16 and 0.80, respectively. The web version developed together was very easy to use. The risk stratification indicated that high-risk patients (risk point > 195.67) were more likely to develop G2+ acute HT [odds ratio (OR) = 2.17, 95% confidence interval (CI): 1.30–3.05].

**Conclusion:**

This nomogram well-predicted the risk of G2+ acute HT during IMRT in cervical cancer after the PBM sparing strategy, and the constructed risk stratification could assist physicians in screening high-risk patients and provide a useful reference for future prevention and treatment strategies for acute HT.

## Introduction

Acute hematologic toxicity (HT) is a common complication during intensity-modulated radiation therapy (IMRT) for cervical cancer. Therefore, many researchers recommend limiting the dose of pelvic bone marrow (PBM) with good results (1–9). Despite this, some patients still developed acute HT during IMRT, but little attention has been paid to it. According to statistics, the risk of cervical cancer grade 2 and higher (G2+) acute HT during chemoradiotherapy ranged from 60% to 90%, (4) and Peters et al. (10) reported that about 74% of patients had G2+ leukopenia. Acute HT may lead to delays and interruptions in treatment, resulting in poorer clinical outcomes (10–12). Therefore, it was necessary to clarify the related factors of acute HT in cervical cancer after the PBM sparing strategy and identify high-risk patients to ensure the successful implementation of the treatment.

At present, personalized prediction models constructed by multiple predictors have been promoted and applied in several fields (13–15). Although many prediction models associated with cervical cancer have been developed, they are rarely used to predict the risk of acute HT (16, 17). Many studies reported that the patient's status [age, body mass index (BMI)], tumor status, and treatment strategies (surgery, chemotherapy, radiotherapy) were possible risk factors causing HT, (8, 9, 16, 18) but a comprehensive and convenient risk assessment system has not been established. A nomogram can predict the probability of acute HT and identify high-risk patients to help clinicians to making rational strategies for avoiding over-or under-treatment.

This study aimed to identify predictors associated with HT and use a web calculator to develop a nomogram for predicting G2+ acute HT in cervical cancer following the PBM sparing strategy. This work can provide a useful reference for future prevention and treatment strategies for acute HT complications.

## Materials and methods

### Patients

Approved by the Institutional Review Board, this study retrospectively analyzed patients with cervical cancer who underwent IMRT at our institution from September 2020 to April 2022. All patients underwent hematology testing 1 week before IMRT (baseline), weekly during IMRT, and 2 weeks after IMRT. In addition, we used the following exclusion criteria: (1) Missing hematology test results; (2) Failure to complete IMRT treatment; (3) Patients with heavy bleeding caused by cervical cancer; (4) No PBM sparing strategy; (5) Lack of necessary follow-up information; (6) Suffering from other cancers or received chemoradiotherapy. All patient data in this study are anonymous.

### Clinicopathologic characteristics

All patients were pathologically diagnosed with cervical cancer. Combining previous studies and factors that may contribute to a decrease in hematocrit Age at diagnosis, underlying disease (hypertension, diabetes, and hyperthyroidism), BMI, pathology type, histological grade, surgery, pre-radiotherapy chemotherapy, pre-radiotherapy HT, and concurrent chemoradiotherapy (CCRT) were selected as potential predictors in this study.

### Treatment

Unless intolerable, the surgery was decided by the International Federation of Gynecology and Obstetrics (FIGO 2018th edition) stage (19). The radiotherapy strategies were divided into IMRT alone and IMRT with simultaneous cisplatin. All patients in this study received 50 Gray (Gy)-50.4 Gy of IMRT in 25–28 fractions of 1.8 Gy-2.0 Gy each. (Using 6-MV X-rays). If receiving CCRT, cisplatin treatment was given in 4–6 cycles at a dose of 40 mg/m^2^ with a maximum dose of 70 mg. The chemotherapy regimen before radiotherapy was albumin paclitaxel 260 mg/m^2^ and cisplatin 75 mg/m^2^ and was given at 3-week intervals two times. Notably, chemotherapy was discontinued when the white blood cell (WBC) count was <2.0 × 10^9^/L, absolute neutrophil count (ANC) < 1.0 × 10^9^/L, and platelets (ALT) <50 × 10^9^/L.

### Pelvic bone marrow sparing strategy

This study established IMRT treatment plans based on the Eclipse 15.6 system and depicted the PBM (including the sacrum, coccyx, and hip bones). After planning, dose-volume histograms (DVHs) were then created and recorded for PBM volumes receiving 10, 20, 30, 40, and 50 Gy (V10, V20, V30, V40, and V50). The PBM dose from brachytherapy is considered negligible (16). Our PBM sparing strategy were V10 < 95%, V20 < 80%, V30 < 50%, V40 < 25%, and V50 < 10%. The target areas for all patients were outlined by two physicians and the same medical physicist designed the IMRT plan.

### Acute hematologic toxicity

Acute HT was graded according to the radiation therapy oncology group (RTOG) acute radiation therapy morbidity scoring criteria, (20) and was defined when one of the four indicators (leukopenia, neutropenia, anemia, and thrombocytopenia) occurred. The lowest counts of WBC, ANC, hemoglobin (Hgb), and PLT were recorded in this study. The study endpoint was G2+ HT (WBC ≤ 3 × 10^9^/L, ANC ≤ 1.5 × 10^9^/L, Hgb ≤ 95g/L, or ALT ≤ 75 × 10^9^/L) observed during IMRT or within 2 weeks after IMRT.

### Statistical analysis

Continuous variables that met normality and variance chi-squared were reported as mean and standard deviation (SD), otherwise as the median and interquartile range (IQR). For categorical variables, counts and percentages were performed.

Three methods were used to screen predictors in our study: univariate and multivariate logistic regression, best subset regression (BSR), and least absolute shrinkage and selection operator (LASSO) regression. Variables with *P* < 0.1 in univariate logistic regression were included in multivariate logistic regression, which selected variables with *P* < 0.05 for modeling. An R-squared maximum was used for the BSR, and the LASSO regression used the minimum mean square error (MSE) plus one standard error (SE) to find a penalty coefficient (lambda, λ) as the variable selection criterion. To avoid underfitting or overfitting, the final model used to develop the nomogram was determined using the minimum Akaike information criterion (AIC). In addition, all predictors were tested for correlation to avoid multicollinearity.

Based on the above screened predictors, a nomogram was developed to predict G2+ acute HT following PBM sparing strategy in cervical cancer, which quantified each variable. The total risk point obtained after summing the points for all variables corresponds to a probability of G2+ acute HT. For ease of use, a web version of the model has been created.

The calibration of the nomogram was assessed using calibration plots and Brier scores, and its discrimination was assessed by the concordance index (C-index) and receiver operating characteristic (ROC) curve. The Brier Score was used to evaluate the overall performance of the model, which combined discrimination and calibration. The closer the calibration curve was to the diagonal and the smaller the Brier score, the better the agreement between the model and the reality. The closer the C-index and the area under the curve (AUC) of ROC were to 1, the better the model's discrimination. Moreover, internal validation was performed with 1000 times of five-fold cross-validation. The clinical usefulness was performed by decision curve analysis (DCA). Patients were divided into low- and high-HT risk groups by calculating the total risk point for all patients based on the nomogram. The criteria for grouping were determined by the optimal cut-off value of the risk point calculated on the ROC. Finally, the risk grouping was validated by a confusion matrix and the differences between the two subgroups of G2+ acute HT were compared.

Statistical analysis of this study was completed by R (version 4.1.0, http://www.rproject.org/). *P* < 0.05 was considered statistically significant.

## Results

### Study population characteristics

A total of 125 patients were pathologically diagnosed with cervical cancer and underwent IMRT. The mean age was 53.3 years (SD = 12.37 years), of which 103 were squamous cell carcinomas, 20 were adenocarcinomas, and 2 were neuroendocrine carcinomas. According to FIGO staging, 55 (44.0%) patients were in stage I, 26 (20.8%) in stage II, 40 (32.0%) in stage III, and 4 (3.2%) in stage IV. Of these patients, 76 (60.8%) received CCRT, and 49 (39.2%) received IMRT alone. In addition, 76 (60.8%) underwent surgery, and 72 (57.6%) received chemotherapy prior to IMRT. Details are shown in Table 1.

**Table 1:** Clinicopathological characteristics of the patient (*N* = 125).

**Characteristics**	**All patients** **(*N* = 125)**	**Non-G2+ acute HT** **(*N* = 38)**	**G2+ acute HT** **(*N* = 87)**
**Age at diagnosis**, ***n*** **(%)**			
≤65	105 (84.00)	28 (73.68)	77 (88.51)
>65	20 (16.00)	10 (26.32)	10 (11.49)
Mean (SD), year	53.30 (12.37)	54.79 (13.60)	52.66 (11.82)
**Chronic diseases**, ***n*** **(%)**			
No	77 (61.60)	18 (47.37)	59 (67.82)
Yes	48 (38.40)	20 (52.63)	28 (32.18)
**BMI**, ***n*** **(%)**			
<18.5	1 (0.80)	0 (0.00)	1 (1.15)
18.5–23.9	56 (44.80)	16 (42.11)	40 (45.98)
≥24	68 (54.40)	22 (57.89)	46 (52.87)
Median (IQR), kg/m^2^	24.19 (22.27, 26.04)	24.14 (22.27, 26.04)	24.29 (22.57, 26.15)
**Pathology**, ***n*** **(%)**			
Squamous carcinoma	103 (82.40)	33 (86.84)	70 (80.46)
Adenocarcinoma	20 (16.00)	5 (13.16)	15 (17.24)
Other	2 (1.60)	0 (0.00)	2 (2.30)
**Histological grade**, ***n*** **(%)**			
I	21 (16.80)	12 (31.58)	9 (10.34)
II–III	104 (83.20)	26 (68.42)	78 (89.66)
**FIGO staging (2018)**, ***n*** **(%)**			
I	55 (44.00)	22 (57.89)	33 (37.93)
II	26 (20.80)	7 (18.42)	19 (21.84)
III	40 (32.00)	9 (23.68)	31 (35.63)
IV	4 (3.20)	0 (0.00)	4 (4.60)
**Surgery**, ***n*** **(%)**			
No	49 (39.20)	18 (47.37)	31 (35.63)
Yes	76 (60.80)	20 (52.63)	56 (64.37)
**Pre-radiotherapy chemotherapy**, ***n*** **(%)**			
No	53 (42.40)	23 (60.53)	30 (34.48)
Yes	72 (57.60)	15 (39.47)	57 (65.52)
**Radiotherapy**, ***n*** **(%)**			
IMRT alone	49 (39.20)	21 (55.26)	28 (32.18)
CCRT	76 (60.80)	17 (44.74)	59 (67.82)
**Pre-radiotherapy HT**, ***n*** **(%)**			
No	41 (32.80)	23 (60.53)	18 (20.69)
Yes	84 (67.20)	15 (39.47)	69 (79.31)
**HT during radiotherapy**, ***n*** **(%)**			
No	38 (30.40)	38 (100.00)	0 (0.00)
Yes	87 (69.60)	0 (0.00)	87 (100.00)

SD, standard deviation; BMI, body mass index; IQR, interquartile range; FIGO, international federation of gynecology and obstetrics; IMRT, intensity-modulated radiation therapy; CCRT: concurrent chemoradiotherapy.

In the present study, 84 (67.20%) patients had G1+ acute HT before IMRT, and 87 (69.60%) patients had G2+ acute HT during IMRT (Table 2). In designing the IMRT plan, we limited the dose to the PBM under the condition that 95% of the prescribed dose covered the planned target volume (PTV). The results showed that acute HT of grades 1–4 during radiotherapy in this study did not correlate significantly with V10, V20, V30, V40, or V50 of PBM (all *P* values > 0.05 in Table 3).

**Table 2:** Acute hematological toxicity before and during radiotherapy in patients with cervical cancer.

**Acute HT grade**	**Leukopenia**	**Neutropenia**	**Anemia**	**Thrombocytopenia**	**Overall HT**
**Grade** **≥1**, ***n*** **(%)**					
Pre-IMRT	61 (48.80)	44 (35.20)	44 (35.20)	10 (8.00)	84 (67.20)
Post-IMRT	106 (84.80)	75 (60.00)	65 (52.00)	33 (26.40)	112 (89.60)
**Grade ≥2**, ***n*** **(%)**					
Pre-IMRT	29 (23.20)	32 (25.60)	15 (12.00)	3 (2.40)	50 (40.00)
Post-IMRT	77 (61.60)	48 (38.40)	25 (20.00)	15 (12.00)	87 (69.60)
**Grade ≥3**, ***n*** **(%)**					
Pre-IMRT	9 (7.20)	22 (17.60)	3 (2.40)	0 (0.00)	26 (20.80)
Post-IMRT	27 (21.60)	24 (19.20)	6 (4.80)	6 (4.80)	39 (31.20)
**Grade = 4**, ***n*** **(%)**					
Pre-IMRT	0 (0.00)	4 (3.20)	1 (0.80)	0 (0.00)	5 (4.00)
Post-IMRT	3 (2.40)	6 (4.80)	0 (0.00)	0 (0.00)	6 (4.80)

HT, hematological toxicity; IMRT, intensity-modulated radiation therapy.

**Table 3:** Univariate analysis of the effect of pelvic bone marrow with different radiation volumes on acute hematological toxicity.

**PBM dose**	**Mean (SD)**	**Grade** ≥**1**		**Grade** ≥**2**		**Grade** ≥**3**		**Grade** = **4**	
		***R* ^2^**	***P*-value**	***R* ^2^**	***P*-value**	***R* ^2^**	***P*-value**	***R* ^2^**	***P*-value**
V10	94.67 (3.36)	0.012	0.3835	0.028	0.1135	0.014	0.2785	<0.001	0.9683
V20	77.37 (8.38)	0.009	0.4575	0.006	0.4692	<0.001	0.8461	0.082	0.0717
V30	41.16 (5.91)	0.016	0.3249	0.001	0.7509	0.001	0.7761	0.057	0.1458
V40	20.72 (4.37)	0.036	0.1442	0.005	0.5086	<0.001	0.9203	0.081	0.0895
V50	6.11 (4.68)	0.008	0.4740	<0.001	0.8479	<0.001	0.9173	0.006	0.6317

PBM, pelvic bone marrow; SD, standard deviation.

### Predictor screening

**Figure 1:**
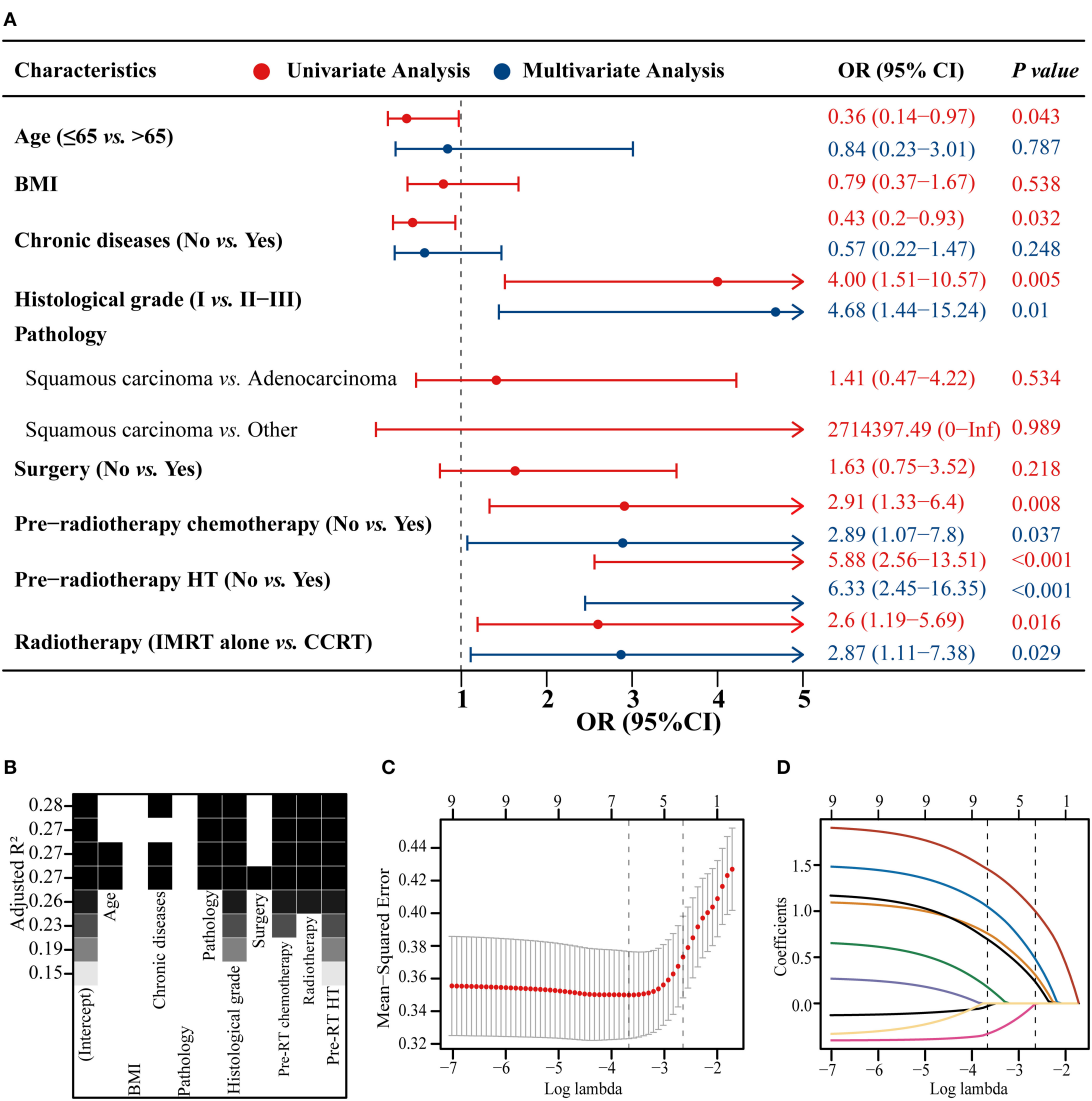
Three methods of screening predictors. Univariate and multivariate logistic regression forest plot **(A)**, best subset regression (BSR) **(B)**, and least absolute shrinkage and selection operator (LASSO) regression plus 10-fold cross-validation **(C,D)**. BMI, body mass index; HT, hematological toxicity; IMRT, intensity modulated radiation therapy; CCRT, concurrent chemoradiotherapy; OR, odds ratio; CI, confidence interval.

**Figure 2:**
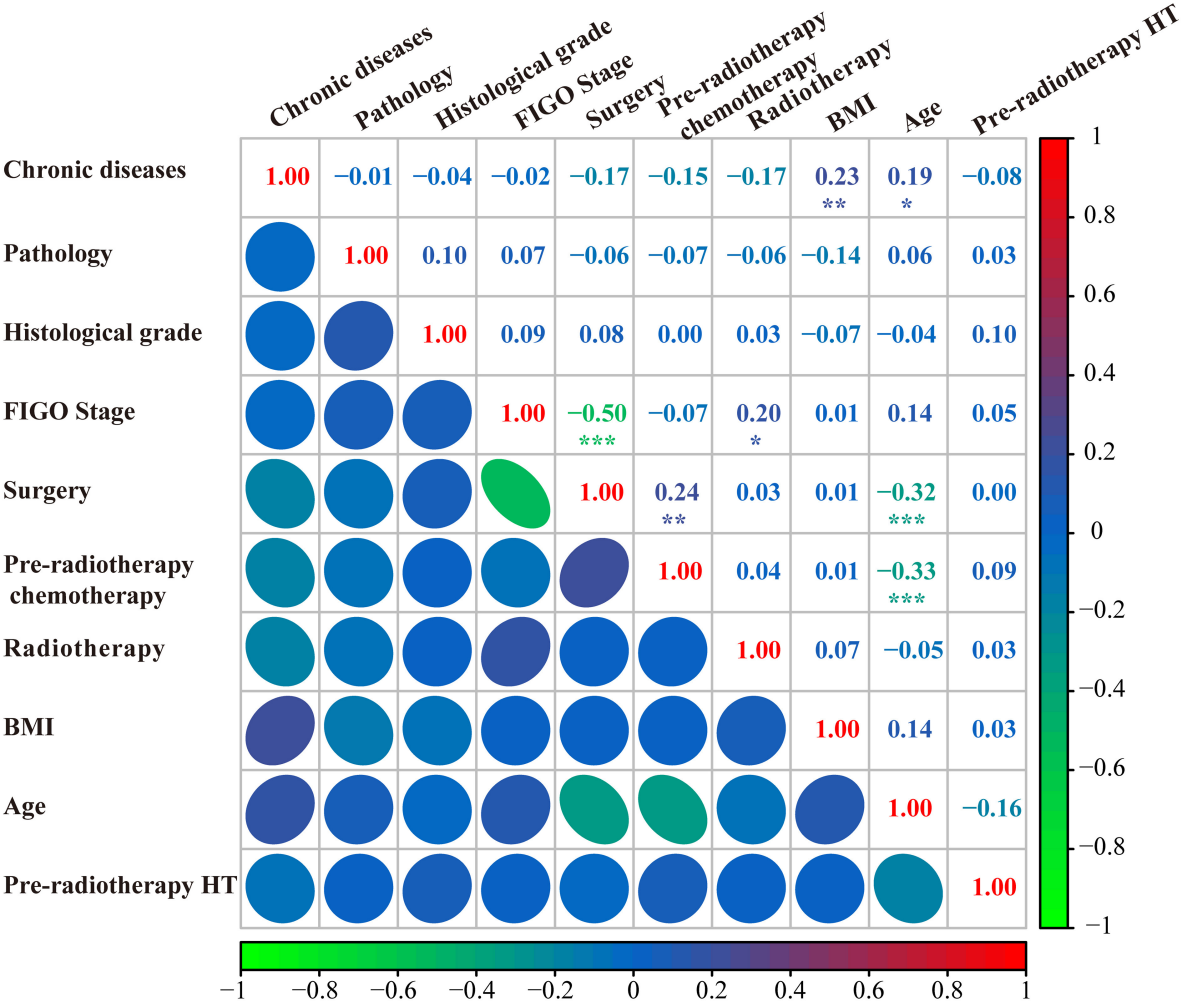
Correlation analysis of all predictors. FIGO, international federation of gynecology and obstetrics; BMI, body mass index; HT, hematological toxicity.

The model constructed with the variables screened by univariate and multifactorial logistic regression has the smallest AIC of 125.49, compared with 126.03 for the BSR model and 126.01 for the LASSO regression (Figure 1). The subsets of variables screened by the three methods are summarized in Table 4. Meanwhile, spearman correlation analysis was performed between all predictors to avoid multicollinearity effects among the predictors (Figure 2). Finally, histological grade (I vs. II–III), pre-radiotherapy chemotherapy (Yes vs. No), pre-radiotherapy HT (Yes vs. No), and radiotherapy (IMRT alone vs. CCRT) were selected as independent predictors to develop the predictive model.

**Table 4:** Univariate and multivariate logistic regression, BSR and LASSO regression for screening predictors.

**Characteristics**	**Univariate and multivariate logistic regression**	**BSR**	**LASSO regression**
Age			
BMI		Ture	
Chronic diseases		Ture	Ture
Pathology			
Histological grade	Ture	Ture	Ture
Surgery			
Pre-radiotherapy chemotherapy	Ture	Ture	Ture
Pre-radiotherapy HT	Ture	Ture	Ture
Radiotherapy	Ture	Ture	Ture
AIC values	125.49	126.03	126.01
Number of predictors	4	6	5

BMI, body mass index; HT, hematological toxicity; AIC, Akaike information criterion; BSR, best subset regression; LASSO, least absolute shrinkage and selection operator.

### Development and validation of the nomogram

**Figure 3:**
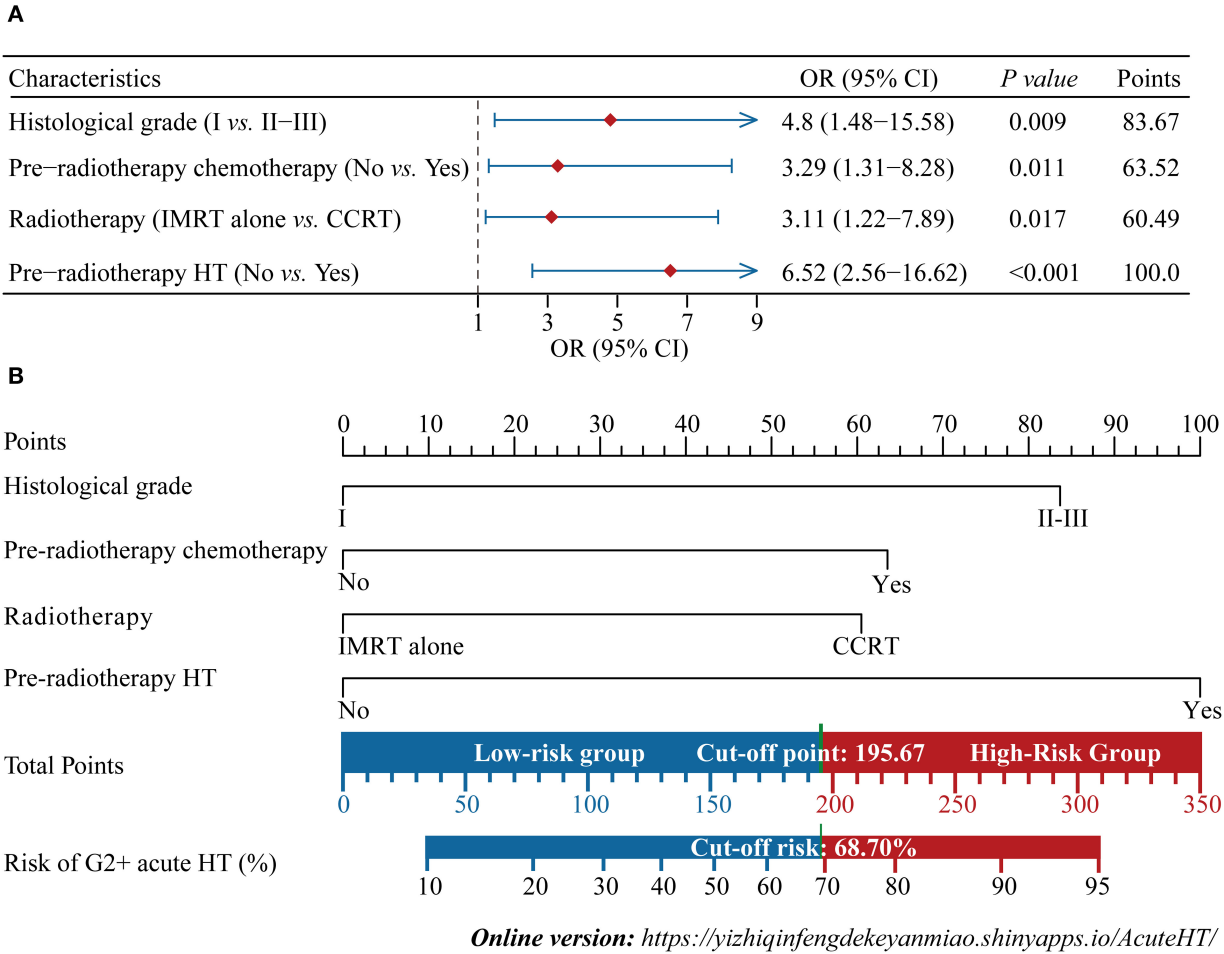
Development of the nomogram. Multivariate logistic regression forest plot for four independent predictors **(A)**, and the nomogram developed based on these predictors **(B)**. IMRT, intensity modulated radiation therapy; CCRT: concurrent chemoradiotherapy; HT, hematological toxicity; OR, odds ratio; CI, confidence interval. G2+, Grade ≥2.

**Figure 4:**
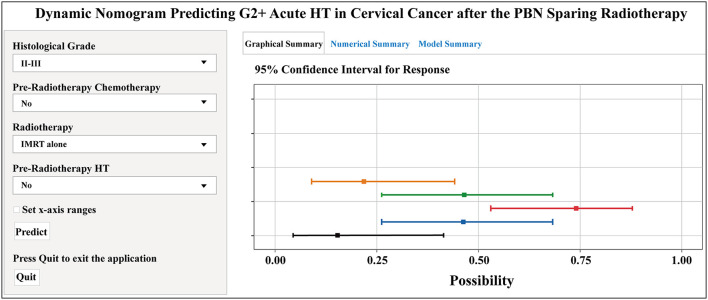
Dynamic nomogram predicting G2+ acute HT in cervical cancer after the pelvic bone marrow (PBM) sparing radiotherapy (https://yizhiqinfengdekeyanmiao.shinyapps.io/AcuteHT/). G2+, Grade ≥2; HT, hematological toxicity.

The forest plot showed the effects of the above four predictors on G2+ acute HT (Figure 3A) and based on these variables, we developed a nomogram (Figure 3B). This model quantified each variable and allowed individualized calculation of the patient's total risk point corresponding to the likelihood of G2+ acute HT. An online version of this nomogram could be found at https://yizhiqinfengdekeyanmiao.shinyapps.io/AcuteHT/ (Figure 4), which automatically calculates HT risk simply by entering patient's information. In addition, the mathematical expression of the HT complication probability was G2+ acute HT (%) = −6.2 × 10^−8^ × points^3^ + 2.8182 × 10^−5^ × points^2^ + 9.3968 × 10^−5^ × points +0.054123045.

**Figure 5:**
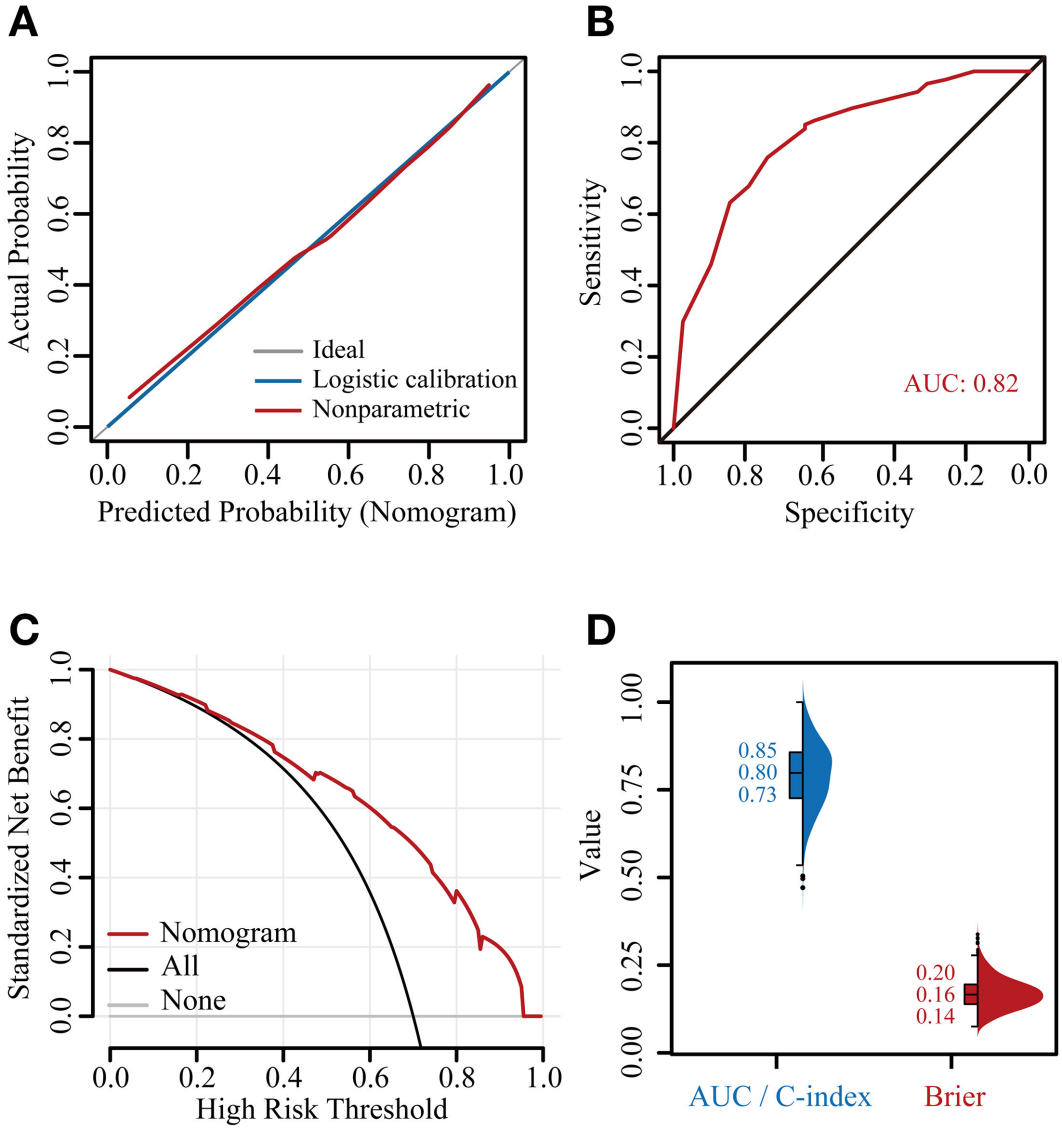
Verification of the nomogram. Calibration plot **(A)**, receiver operating characteristic (ROC) curve **(B)**, decision curve analysis (DCA) curve **(C)**, and 1000 times five-fold cross-validated C-indexes and Brier scores **(D)**. AUC, area under the curve; C-index, concordance index.

The calibration curve showed good consistency between the risk of G2+ acute HT predicted by the nomogram and the actual risk, close to the 45° line, and the Brier score was also very low at 0.15 (Figure 5A). The C-index and AUC of the model were both 0.82 [confidence interval (CI): 0.74–0.90] (Figure 5B). The DCA showed that, for the same threshold probability, more net gain could be obtained using the nomogram (Figure 5C). When the threshold probability of G2+ acute HT was 0.06–0.95, the net benefit of applying the nomogram (range: 6%−68%) was significantly higher than that of the “no intervention” and “full intervention” strategies. To further evaluate the performance of the model, we performed 1000 times five-fold cross-validation on the C-index (equal to AUC) and Brier score, where the median value of the C-index was 0.80 (IQR: 0.73–0.85), and the Brier score was 0.16 (IQR: 0.14–0.20) (Figure 5D).

### Application of the nomogram

**Figure 6:**
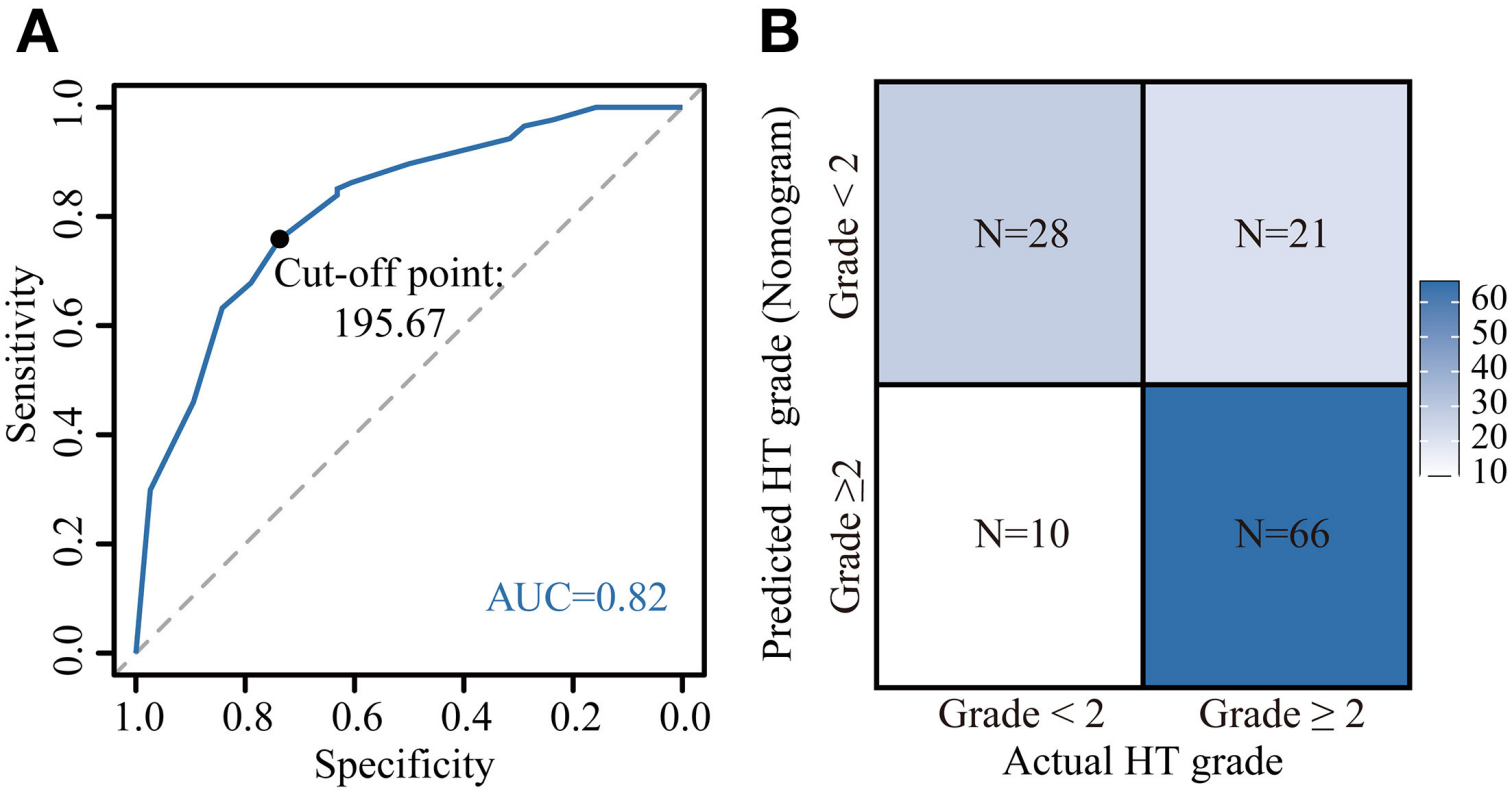
Application of the nomogram. Risk stratification constructed by calculating the optimal cut-off value of the total point by the receiver operating characteristic (ROC) curve **(A)**. [A total point below 195.67 was included in the low-risk group (risk of acute HT < 2), otherwise the high-risk group (risk of acute HT ≥2)]. Confusion matrix for assessing differences between predicted risk and actual risk of G2+ acute HT **(B)**. HT, hematological toxicity.

Based on the nomogram quantification of risk, we calculated the total risk points for 125 patients and stratified them by finding the best cut-off score according to the ROC (Figure 6A). The low-risk group (39.2%, 49/125) had a risk point of ≤ 195.67 (risk probability ≤ 68.7%), while the others were the high-risk group (60.8%, 76/125). Meanwhile, Patients in the high-risk group were 2.17 (95% CI: 1.30–3.05) times more likely to develop G2+ acute HT complications than those in the low-risk group. The confusion matrix showed an accuracy of 0.75 (95% CI: 0.67–0.82) for this risk stratification (Figure 6B).

## Discussion

Despite the PBM sparing strategy, some cervical cancer patients suffered from acute HT during IMRT. In this work, we developed a nomogram with an online version to predict G2+ acute HT risk for these patients. Meanwhile, the risk stratification constructed based on the model could well-identify high-risk patients and help clinicians make more rational decisions on the prevention and management of HT complications.

There were many factors that lead to acute HT in patients with cervical cancer, chemotherapy, surgery, patient's physical condition, and tumor status (8, 9, 16, 18). It was well-known that the pelvis contains approximately 50% of the human hematopoietic PBM, which was frequently exposed to the radiotherapy target of cervical cancer (3, 16). Moreover, PBM was extremely sensitive to radiation, and even low doses could adversely affect it (21, 22). IMRT was of increasing interest as it could reduce the dose of PBM and the risk of acute HT (1, 3–5, 7, 23, 24). Hui et al. (24) concluded that three-dimensional conformal radiotherapy (3DCRT) had more severe G2+ leukopenia and neutropenia than IMRT and recommended the use of IMRT to reduce the volume of PBM irradiation and the incidence and severity of acute HT. In the IMRT era, many studies have explored appropriate PBM sparing strategies. Mutyala et al. (5) recommended V10 < 95%, V20 < 80% and V30 < 64% for PBM. Huang et al. (2) recommended V10 ≤ 85%, V20 ≤ 65%, and V30 ≤ 45%. A prospective randomized controlled trial by Huang et al. (1) recommended V40 < 28% for PBM to reduce the risk of G2+ acute HT. RTOG 0418 reported that V40 > 37% of PBM was associated with a higher incidence of G2+ toxicity in cervical cancer receiving CCRT [odds ratio (OR) = 4.5, 95% CI: 1.2–17.4, *P* = 0.029] (25). The PBM sparing strategy used at our institution (V10 < 95%, V20 < 80%, V30 < 50%, V40 < 25%, and V50 < 10%) also had no effect on acute HT in grades 1–4 at IMRT. Unfortunately, there were still many patients who suffered from acute HT. Previous studies have suggested that HT was not only related to PBM irradiation but may also be related to the trauma of surgery, chemotherapy, baseline blood parameters, and individual status (1, 8, 18). Therefore, improving the dose limit of PBM alone would still miss some high-risk HT patients. Based on these, we identified other factors contributing to HT under the condition of limited PBM dose.

As a statistical model, the nomogram can combine multiple predictors to achieve individualized predictions (26). To ensure the model's goodness-of-fit, we used three methods to screen for prognostic factors. Ultimately, histological grade, pre-radiotherapy chemotherapy, pre-radiotherapy HT, and CCRT were used as independent prognostic factors to predict G2+ acute HT to develop the nomogram. The high histological grade may be associated with poorer prognosis in cervical cancer, which promoted adverse event complications (27). So, we included histologic grade in this model although no molecular mechanism associated with HT was found. In addition, any period of chemotherapy or pre-radiotherapy HT may result in patients suffering from HT during IMRT (5, 8, 16, 18). Finally, the correlation heat map confirmed that there was no correlation among these four predictors, thus avoiding multicollinearity. This nomogram was rigorously evaluated and internally cross-validated and showed good predictive performance. There were few reports on the use of HT to predict cervical cancer. Although Mutyala et al. (5) reported an HT probability prediction nomogram and proposed a PBM dose constraint for the IMRT in cervical cancer, the model still lacked some clinical factors. Our model may be the first tool to predict the risk of HT during IMRT after limiting the PBM dose. In addition, we have developed a web version of this model so that physicians and patients can easily use it.

The ability to accurately identify patients at different risks was equally important, as it was a prerequisite for the development of treatment strategies (14, 26). Therefore, we constructed a risk stratification in which two subgroups showed significant differences. Unlike previous nomograms that only showed the percentage of events that occurred, this stratification was able to classify patients into high- or low-HT subgroups. This made it easier and more intuitive to assist physicians in managing patients and making sound treatment decisions.

However, the study has some limitations. First, retrospective studies were inevitably subject to selection bias. Secondly, the nomogram we developed has not been externally validated by other medical institutions. Third, the accuracy of risk stratification was 75.2%, which may be related to our small sample size. Fourth, as treatment techniques improve, acute HT events may decline and the predictive power of the nomogram may be compromised.

## Conclusion

Following the PBM sparing strategy, we developed the first nomogram for individualized prediction of the risk of G2+ acute HT in patients with cervical cancer. This model was quite accurate and had an easy-to-use web-based version. The risk stratification constructed from the nomogram could help physicians screen high-risk patients when preventing acute HT complications. In addition, this nomogram still requires external validation.

## Data availability statement

The raw data supporting the conclusions of this article will be made available by the authors, without undue reservation.

## Ethics statement

The studies involving human participants were reviewed and approved by Institutional Review Board of Weifang People's Hospital. The patients/participants provided their written informed consent to participate in this study.

## Author contributions

XM and YL designed the study. XM, NW, and YL performed the study and analyzed the data. XM wrote the manuscript. XC and YG provided the expert consultations and clinical suggestions. DK, ZZ, and XC conceived of the study, participated in its design, and coordination. NW, XC, and YG helped to draft the manuscript. All authors contributed to the article and approved the submitted version.

## Conflict of interest

The authors declare that the research was conducted in the absence of any commercial or financial relationships that could be construed as a potential conflict of interest.

## Publisher's note

All claims expressed in this article are solely those of the authors and do not necessarily represent those of their affiliated organizations, or those of the publisher, the editors and the reviewers. Any product that may be evaluated in this article, or claim that may be made by its manufacturer, is not guaranteed or endorsed by the publisher.

## Abbreviations

HT: hematologic toxicity
IMRT: intensity-modulated radiation therapy
PBM: pelvic bone marrow
G2+: grade 2 and higher
BMI: body mass index
CCRT: concurrent chemoradiotherapy
FIGO: the International Federation of Gynecology and Obstetrics
Gy: Gray
WBC: white blood cell
ANC: absolute neutrophil count
Hgb: hemoglobin
PLT: platelets
RTOG: the radiation therapy oncology group
DVH: dose-volume histogram
SD: standard deviation
IQR: interquartile range
BSR: best subset regression
LASSO: least absolute shrinkage and selection operator
SE: standard error
AIC: Akaike information criterion
C-index: concordance index
ROC: receiver operating characteristic
AUC: the area under the curve
DCA: decision curve analysis
CI: confidence interval.
